# Incidence and risk factors of anaemia among people on antiretroviral therapy in Harare

**DOI:** 10.4102/sajhivmed.v25i1.1605

**Published:** 2024-08-30

**Authors:** Linda A. Mandikiyana Chirimuta, Tinei Shamu, Cleophas Chimbetete, Chérie Part

**Affiliations:** 1 Newlands Clinic, Harare, Zimbabwe; 2 Department of Public Health, Environments and Society, Faculty of Public Health and Policy, London School of Hygiene and Tropical Medicine, London, United Kingdom; 3 Institute of Social and Preventive Medicine, University of Bern, Bern, Switzerland; 4 Graduate School of Health Sciences, University of Bern, Bern, Switzerland

**Keywords:** HIV, anaemia, antiretroviral therapy, zidovudine, antiretroviral therapy toxicities, haematology, anaemia

## Abstract

**Background:**

Anaemia is associated with reduced quality of life and increased mortality risk among people living with HIV (PLHIV). Although antiretroviral therapy (ART) reduces the prevalence of anaemia, some patients remain at risk after commencing ART.

**Objectives:**

We estimated the incidence of anaemia after ART commencement and identified associated risk factors.

**Method:**

We analysed outpatient records at Newlands Clinic, Harare, Zimbabwe. Patients (≥ 5 years old) who were commenced on ART between January 2016 and December 2020 were included and were followed up for up to 2 years. Patients with anaemia at ART commencement and women who were pregnant at any time during follow-up were excluded. Cox proportional hazards regression was used to assess independent risk factors for anaemia.

**Results:**

During the study, 1110 patients ≥ 5 years old were commenced on ART with a prevalence of anaemia of 40.0%. Five hundred and twenty-nine patients met the inclusion criteria and were followed up for 823.7 person-years. The median age was 36.1 years and 290 (58.4%) were female. The incidence rate of anaemia after ART commencement was 176.1 per 1000 person-years (95% confidence interval [CI]: 149.6–207.2). Females (aHR: 2.09; 95% CI: 1.46–3.00, *P* < 0.001), zidovudine use (aHR: 3.50 96% CI: 2.14–5.71, *P* < 0.001), age 5–12 years or > 50 years, and the presence of World Health Organization stage III/IV disease (aHR: 2.19; 95% CI: 1.14–5.71, *P* = 0.019) had higher odds of developing anaemia.

**Conclusion:**

The incidence of anaemia after ART commencement was high. Female sex, zidovudine use, age and the presence of stage III/IV disease were independent risk factors for anaemia. Clinicians should screen PLHIV on ART regularly for anaemia.

**What this study adds:** This study found a high incidence of anaemia among PLHIV after commencing ART and identified several risk factors.

## Introduction

Anaemia is the most common haematological abnormality affecting people living with HIV (PLHIV).^1,2^ In PLHIV, anaemia serves as an independent prognostic indicator, and is associated with disease progression, reduced quality of life, and mortality.^2,3,4,5,6^ Although antiretroviral therapy (ART) has reduced the prevalence of anaemia among PLHIV,^7,8,9^ prevalence remains high compared to HIV-negative individuals.^1,9,10,11,12^ In high-income countries, more than 30% of PLHIV have anaemia.^2,13^ It is estimated that 27% of women and 15% of men in the general population of Zimbabwe are anaemic;^14^ however, the relative burden of anaemia among PLHIV is unknown.

Multiple factors have been proposed in the aetiology of anaemia in HIV infection, including: (1) blood loss in the gastro-intestinal tract, which is associated with neoplastic disease and opportunistic infections; (2) decreased red cell production from infiltration of the bone marrow by HIV and opportunistic infections; (3) adverse drug effects; (4) ineffective red cell production caused by the nutritional deficiencies commonly seen in PLHIV; and (5) HIV-related red cell destruction.^15^

Risk factors commonly associated with anaemia in HIV include advanced HIV disease, low CD4 count, high viral load, the presence of an opportunistic infection, female sex, and lower body mass index (BMI).^10,15^ Although the risk of anaemia in PLHIV is reduced by taking ART,^5,6,7,16^ there are data showing that that some individuals remain anaemic, and other individuals develop anaemia after commencing ART. For those who develop anaemia, the incidence is highest during the first few months on ART.^17^ However, current knowledge comes from cross-sectional studies conducted in high-income countries, where the prevalence of HIV is relatively low. In an era of widespread ART availability in sub-Saharan African countries, studies are needed to inform the continued care of PLHIV, particularly within primary care facilities, and to identify patients at greatest risk of comorbidities in order to target limited resources for screening and intervention.

This study set out to understand the incidence, aetiology, and associated risk factors of anaemia among a cohort of PLHIV on ART in Zimbabwe in order to provide recommendations to reduce the adverse impacts of HIV-associated anaemia.

## Research methods and design

### Study design and setting

This was a retrospective cohort study conducted at Newlands Clinic, an outpatient HIV management centre in an urban area of Harare, Zimbabwe. The clinic was founded in 2004 and operates in a public-private partnership with the Ministry of Health and Childcare of Zimbabwe to provide comprehensive treatment services to PLHIV. Newlands Clinic currently cares for 7650 patients, predominantly residents of Harare, but also PLHIV from other areas of Zimbabwe.

We included all PLHIV, aged 5 years and older, who were initiated on any ART regimen between January 2016 to December 2020. We abstracted records of patients with at least two full blood counts (FBC) and followed them up for 2 years on ART, or until the end of the study (30 April 2021), whichever came first. The follow-up period was kept at a maximum of 2 years because exposures were measured at time of ART commencement only. At Newlands Clinic, all PLHIV are eligible for ART commencement. Between 2016 and 2019, the first-line regimen used in adults was a fixed-dose combination of tenofovir, lamivudine and efavirenz, as per Zimbabwean national guidelines; in 2019, tenofovir, lamivudine and dolutegravir was rolled out as the first-line ART regimen. The first-line ART regimen used in children was zidovudine, lamivudine and nevirapine. Routine FBCs were typically measured at baseline (the time of ART commencement), 12 weeks after ART commencement, and every 6 months thereafter. Anaemia was defined according to the World Health Organization (WHO) age- and sex-specific reference ranges of haemoglobin^18^ (Table 1). We excluded participants who had anaemia at commencement of ART and women who were pregnant at any time during the follow-up period.

**TABLE 1:** World Health Organization definition of anaemia criteria by haemoglobin concentration.

Population	Age group (years)	No anaemia (g/dL)	Anaemia		
			Mild (g/dL)	Moderate (g/dL)	Severe (g/dL)
Children	5–11	≥ 11.5	11.0–11.4	8.0–10.9	< 8.0
Children	12–14	≥ 12.0	11.0–11.9	8.0–10.9	< 8.0
Non-pregnant women	≥ 15	≥ 12.0	11.0–11.9	8.0–10.9	< 8.0
Men	≥ 15	≥ 13.0	11.0–12.9	8.0–10.9	< 8.0

*Source*: Adapted from WHO. Haemoglobin concentrations for the diagnosis of anaemia and assessment of severity [homepage on the Internet]. World Health Organization; 2011 [cited 2021 February 22]. Available from: https://iris.who.int/handle/10665/85839

Data from electronic patient medical records, including demographic and clinical information, were abstracted using customised queries written in Microsoft SQL Server Management Studio version 18, using the defined inclusion and exclusion criteria.

Routinely collected data at Newlands Clinic are managed by a data quality manager who routinely checks the data quality and integrity. The process includes real-time data monitoring using automated checks that analyse a range of data characteristics including missing data (e.g. diagnoses and treatment end dates), parameters inconsistent with life (e.g. unrealistic vital signs and laboratory results), and unlikely date ranges (e.g. dates of birth and diagnosis). Any errors identified are brought to the attention of the responsible staff member with the primary data, and the query is closed after successful resolution. In addition, Newlands Clinic’s participation in the International Epidemiologic Database to Evaluate AIDS (IeDEA) collaboration acts as an independent data review process with numerous tests being conducted on each data set submitted on an annual basis. In the current study, similar tests were also performed following data abstraction, namely data type consistency checks and manual verification of records with extremely low haemoglobin measurements (< 5 g/dL).

### Data analysis

All statistical analyses were undertaken in Stata version 13.1 (StataCorp LP, College Station, Texas, United States). Descriptive statistics (frequencies, proportions, median, interquartile range) were used to describe baseline characteristics of the cohort (baseline was defined as the date on which ART commenced).

The proportion of those who had anaemia at ART commencement (baseline) was calculated and those who had anaemia were further described according to their mean corpuscular volume (MCV) parameters. Microcytic anaemia was defined as anaemia with red cell MCV < 80 femtolitres (fL), macrocytic anaemia was defined as anaemia with red cell MCV > 100 fL, and normocytic anaemia was defined as anaemia with red cell normal MCV 80 fL – 100 fL. Incidence of anaemia was calculated as the number of events divided by the follow-up time per 1000 person-years.

Time-to-event (survival) analysis was conducted to estimate time from ART commencement (start date) to first episode of anaemia (event). Administrative censoring was applied at 104 weeks following ART commencement, or on 30 April 2021 (whichever came first). Right-censoring was also applied to patients who were lost to follow-up, died, transferred out of care, or who discontinued ART.

Survival probabilities were estimated using the Kaplan–Meier method and plotted against time. Here, we stratified by: patient sex (male, female); age (5–12 years, 13–24 years, 25–50 years, > 50 years); HIV viral load (< 10 000 copies/mL, ≥ 10 000 copies/mL); zidovudine exposure (yes, no); chronic kidney disease, defined as stage 3+ (patients with glomerular filtration rate < 60 mL/min per 1.73 m^2^ for 3 months or more)^19^ (present, absent); and weight (not underweight, underweight [defined as low BMI using WHO BMI age-specific reference ranges^20^]). A low BMI for participants aged 5–19 years is less than or equal to −2 standard deviations, using WHO z-scores, and a low BMI for participants aged ≥ 20 years is less than 18.5 kg/m^2^. Differences between survival curves were formally tested using log-rank tests.

Univariable Cox proportional hazards regression analysis was used to calculate hazard ratios (with 95% confidence intervals [CI]) and to identify possible risk factors for anaemia. Hazard ratios were then adjusted for confounding variables, and independent risk factors were identified, using multivariable Cox regression. Sex and age are known confounders of anaemia and so were added a priori to the multivariable model. Other covariates were added to the model following a forward stepwise process. Those variables that had a significant effect on the adjusted hazard ratio were retained in the final model. The proportional hazards assumption was tested for each variable by conducting a formal proportional hazards test based on Schoenfeld residuals.

### Ethical considerations

Ethical approval for this study was granted by the Medical Research Council of Zimbabwe, approval number MRCZ/E/364. Newlands Clinic is part of the IeDEA–Southern African Region (IeDEA-SA). The IeDEA study was approved by the local ethics committee, Medical Research Council of Zimbabwe (reference number MRCZ/B/2064). Patients whose data were used in this study gave informed written consent, which allowed for the data collected during their routine care to be used for the IeDEA-SA study. Participants were not required to sign consent for this secondary data analysis. Full permission to use and analyse the data from the primary study for the purposes of this research study was given in writing from the principal investigator of the primary study. Data were extracted and accessed on 12 March 2024 for research purposes. Patient identification was removed from the data set before being shared with the researcher and the electronic data transfer was encrypted.

## Results

### Study population and baseline characteristics

**FIGURE 1:**
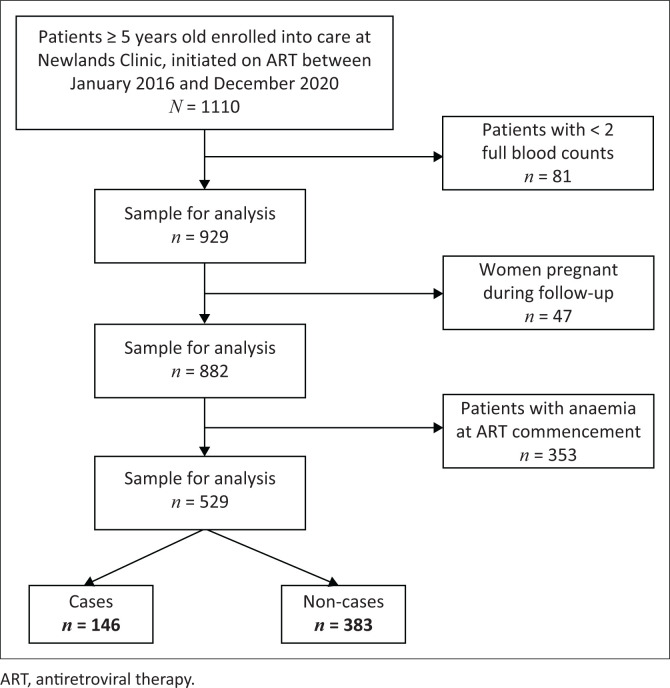
Study flow diagram.

A total of 1110 patients, 5 years old and above, were enrolled in care at Newlands Clinic and started on ART between January 2016 and 31 December 2020. Of these, 81 were excluded for having less than two FBCs, 47 were excluded for being pregnant during follow-up, and 353 for having anaemia at baseline, leaving an eligible sample of 529 participants (Figure 1). The 529 participants were followed up from the time of ART commencement for 823.7 person-years, with a median follow-up time of 104 weeks (interquartile range [IQR]: 56.9–104.0). At the time of ART commencement, the median age was 36.1 years (IQR: 27.0–44.6) and 58.4% of the participants were female. Only 24 participants (4.5%) had a WHO stage III or IV defining illness. The median viral load at the start of ART was 38 684 copies/mL (IQR: 7864–121 329) and the median CD4 count, 313 cells/µL (IQR: 172–463). Table 2 summarises the baseline characteristics of the participants and the incidence of anaemia per characteristic.

**TABLE 2:** Participant characteristics at antiretroviral therapy commencement and incidence of anaemia.

Variables	Total participants (*N*)	Incident anaemia		Person-years at risk	Incidence rate per 1000 person-years	95% CI
		*n*	%			
**All individuals**	-	146	27.6	823.7	176.1	149.6–207.2
**Sex**						
Male	239	46	19.2	392.0	117.4	88.0–156.7
Female	290	100	34.5	431.2	231.6	190.4–281.8
**Age (years)**						
5–12	20	13	65.0	16.6	785.4	456.0–1352.6
13–24	92	28	30.4	137.8	203.1	140.3–294.2
25–50	357	90	25.2	570.9	157.7	128.2–193.8
> 50	60	15	25.0	98.4	152.4	91.9–252.9
**Viral load (copies/mL)** †						
< 10 000	145	39	26.9	213.0	183.1	133.8–250.6
≥ 10 000	378	106	28.0	600.5	176.5	145.9–213.6
**CD4 count (cells/µL)** ‡						
≥ 200	365	102	27.9	570.1	178.0	147.4–217.2
< 200	365	43	11.8	247.4	173.8	128.9–234.4
**Zidovudine**						
Not exposed	492	120	24.4	785.8	152.7	127.7–182.6
Exposed	37	26	70.3	37.8	687.5	468.1–1009.7
**Weight**						
Not underweight	391	104	26.6	594.4	175.0	144.4–212.0
Underweight	138	42	30.4	229.3	183.2	135.4–218.0
**WHO clinical stage**						
Stage I or II	505	136	26.9	791.1	171.9	145.3–203.4
Stage III or IV	24	10	41.7	32.6	306.8	165.1–570.2
**Chronic kidney disease stage 3+**						
No	519	143	27.6	807.7	177.1	150.3–208.6
Yes	10	3	30.0	187.5	187.5	60.5–581.4

WHO, World Health Organization; CI, confidence interval.

† , 6 missing values;

‡ , 4 missing CD4 count values.

### Anaemia incidence and characterisation of anaemia

The overall incidence rate of anaemia in this cohort was 176.1 per 1000 person-years (95% CI: 149.6–207.2), with 27.6% (*n* = 146) individuals developing anaemia during the follow-up period. The median time to developing anaemia after starting ART was 48.1 weeks (IQR: 24.1–91.5).

Of those with incident anaemia, 79.5% (*n* = 116) had normocytic anaemia, 13.7 % (*n* = 20) had macrocytic anaemia, and 6.8% (*n* = 10) had microcytic anaemia.

### Risk factors for anaemia

**FIGURE 2:**
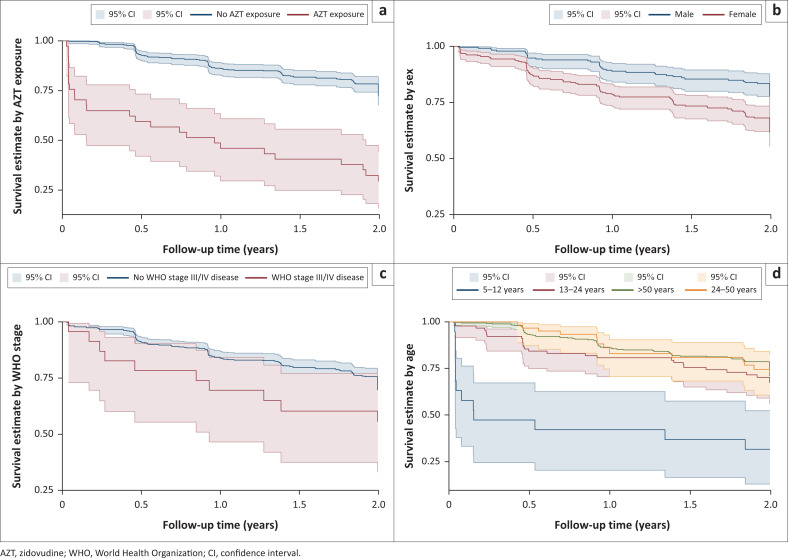
Kaplan–Meier survival estimates showing incident anaemia over time, by (a) exposure to zidovudine (log-rank *P*-value < 0.001), (b) patient sex (log-rank *P*-value < 0.0001), (c) WHO stage (log-rank *P* = 0.079), and (d) age group (log-rank *P*-value < 0.001).

The probability of developing anaemia differed by sex (log-rank *P* < 0.001), age (log-rank test *P <* 0.001), and zidovudine exposure (log-rank *P* < 0.001). There was suggestive evidence that the probability of developing anaemia differed by WHO stage (log-rank test *P* = 0.079). Kaplan–Meier curves, stratified by patient characteristics, showed that female sex, young age (5–12 years), and exposure to a zidovudine-containing ART regime had greater probability of developing anaemia after commencing ART (see Figure 2). Weight (underweight vs not underweight), CD4 count (≥ 200 vs < 200) and chronic kidney disease stage 3+ (present, absent) were not associated with developing anaemia (*P* = 0.738, *P* = 0.868, and *P* = 0.925, respectively).

Univariable and multivariable Cox regression is shown in Table 3. On univariable analysis female sex and exposure to zidovudine were associated with a greater risk of incident anaemia. Age was a protective factor, with the HR decreasing as participants’ age increased. After adjusting for confounders on multivariable analysis the factors independently associated with a greater risk of incident anaemia were female sex, zidovudine exposure, and having WHO stage III or IV disease at baseline. The risk of incident anaemia reduced with increasing age from 13–50 years, with patients 5–12 years and those > 50 years at greatest risk. Viral load above 10 000 copies, being underweight and chronic kidney disease stage 3+ were not independently associated with incident anaemia. The variable CD4 count was excluded from the final model due to collinearity with WHO stage.

**TABLE 3:** Crude and multivariable analysis of anaemia incidence.

Variables	Crude analysis			Multivariable analysis		
	Hazard ratio	95% CI	*P*	Hazard ratio	95% CI	*P*
**Sex**	-	-	< 0.001	-	-	< 0.001
Male	1.00	Reference	-	1.00	Reference	-
Female	2.98	1.40–2.81	-	2.09	1.46–3.00	-
**Age (years)**			< 0.001*			< 0.001*
5–12	1.00	Reference	-	1.00	Reference	-
13–24	0.25	0.13–0.48	-	0.43	0.20–0.92	-
25–50	0.20	0.11–0.35	-	0.34	0.17–0.64	-
> 50	0.19	0.09–0.40	-	0.25	2.14–5.72	-
**Viral load (copies/mL)**	-	-	0.780	-	-	0.871
< 10 000	1.00	Reference	-	1.00	Reference	-
≥ 10 000	0.95	0.66–1.37	-	0.97	0.65–1.44	-
**Zidovudine exposure**	-	-	< 0.001	-	-	< 0.001
Not exposed	1.00	Reference	-	1.00	Reference	-
Exposed	4.64	3.03–7.09	-	3.50	2.14–5.71	-
**Underweight**	-	-	0.812	-	-	0.265
Not underweight	1.00	Reference	-	1.00	Reference	-
Underweight	1.04	0.73–1.50	-	1.23	0.85–1.79	-
**WHO stage III/IV disease**	-	-	0.081	-	-	0.019
No WHO stage III/IV disease	1.00	Reference	-	1.00	Reference	-
WHO stage III/IV disease	1.77	0.93–3.40	-	2.19	1.14–4.23	-
**Chronic kidney disease stage 3+**	-	-	0.917	-	-	0.952
No chronic kidney disease	1.00	Reference	-	1.00	Reference	-
Chronic kidney disease	1.06	0.34–3.33	-	1.03	0.31–3.52	-

Note: *P*-values were obtained using the Chi-square test.

WHO, World Health Organization; CI, confidence interval.

* , *P*-value obtained using likelihood ratio test.

## Discussion

This was one of the few longitudinal studies in a low-income country in sub-Saharan Africa to estimate the incidence of anaemia in patients who commence ART. In this cohort, we observed a high incidence of anaemia among PLHIV, with one in four participants developing anaemia within 2 years of commencing ART. The median time to developing anaemia was close to 1 year (48.1 weeks) after starting ART. Our findings are consistent with a previous study in Ethiopia which found a very high incidence of anaemia, of 353/1000 person-years, in patients who were on ART in Addis Ababa.^17^ Another study done in South Africa found the incidence of anaemia after the first 3 months on ART to be 34.7/1000 person-years on ART, which was lower than what we found in our cohort.^9^ A similar study to ours, done in the United States, found a lower incidence of anaemia, of 19.5/1000 person-years.^21^ This may be because participants in this study may have been commenced on ART with less advanced HIV, among other contributing factors.

Anaemia may be subcategorised morphologically according to the red blood cell MCV, into microcytic, macrocytic, and normocytic. Clinicians often use the MCV of red cells to help determine what may have caused the anaemia in an anaemic individual.^22^ HIV-associated anaemia has been described to be normocytic, similar to that of chronic disease.^3,17,22^ Macrocytic anaemia has been associated with the use of zidovudine.^3,17,22^ The predominant type of anaemia observed in our study was normocytic, which is consistent with Adewuyi et al.’s findings from the pre-ART era in Zimbabwe.^19^ This suggests that the aetiology of the anaemia is chronic disease, in most cases, rather than other aetiologies.

Risk factors for anaemia in this cohort were female sex, zidovudine use, WHO stage III and IV disease, and younger age or age > 50 years, which are previously well documented.^1,21,23,24^ Anaemia is a known adverse effect of zidovudine, and females are already known to be prone to anaemia because of menstrual blood loss and childbearing. Contrary to literature,^5,10,11,12,15,25,26^ advanced HIV disease, being underweight, and having a high viral load at baseline were not significant risk factors for developing anaemia. This may have been because participants who had prevalent anaemia at baseline were excluded from the cohort.

Anaemia is a predicter of low quality of life and disability due to fatigue and its impact on physical performance and cognition.^6,27^ The impact of incident anaemia on these participants’ quality of life is unknown and would be important to investigate in future research.

While FBC monitoring may not be available in primary healthcare facilities offering ART, it is important for healthcare workers, including primary care nurses prescribing ART, to be able to identify clinical signs and symptoms of anaemia so that they can refer upwards for further investigation and management. Clinicians should consider patients of female sex, younger age, and with exposure to zidovudine to be at high risk of incident anaemia. Zidovudine has been phased out as a component of first- and second-line ART regimens in Zimbabwe. One of the reasons for phasing out zidovudine is its toxicity; however, it is still used in third-line regimens and salvage therapy, as guided by resistance testing.^28^

This study has several strengths, including the cohort design. Few cohort studies have been conducted on anaemia among PLHIV in Southern Africa. Most studies that have been done were cross-sectional, prevalence studies with no follow-up period. This is the first study from Zimbabwe looking at incident anaemia among PLHIV in this era of widespread availability of ART. There were few missing data points, and any data that were missing were missing at random, thereby minimising exclusion bias.

This study also has several limitations. We used routinely collected data, which may contain errors that are difficult to identify with tools currently in use at Newlands Clinic. Specifically, current tools may miss inconsistencies in laboratory results or vital signs, leading to inaccurate estimation of the incidence of anaemia. To address this, we employed a manual verification process focused on records with extremely low haemoglobin measurements. Although we recognise that manual verification is less sensitive compared to more sophisticated cross-index system validation methods, this approach was necessitated as we did not have access to advanced tools integrated with the clinic’s existing database systems. While some random transcription errors may have remained unidentified in our data set, we do not expect that they introduced any significant bias after the verification and rigorous data cleaning processes employed at clinic level and in this study. Immune status, viral load and BMI were only measured at baseline and, therefore, might have changed by the time patients had developed anaemia. Further, results may be difficult to generalise to the non-urban health centres of Zimbabwe as Newlands Clinic provides care to a predominantly urban population and people from rural communities may be under-represented. However, our findings are likely generalisable to primary healthcare centres in urban areas.

## Conclusion

The incidence of anaemia was high among PLHIV in Zimbabwe, with one in every four participants developing anaemia, more than half of whom developed anaemia within the first year of ART commencement. Female sex and zidovudine use, younger age, and WHO stage III and IV disease were the most important risk factors for developing anaemia. We recommend that clinicians be trained to detect signs and symptoms of anaemia, and to refer patients appropriately for investigation and further management. Clinicians should maintain a high index of suspicion for incident anaemia among females and those on zidovudine, especially during the first 2 years on ART. Further studies are needed to ascertain the impact of incident anaemia on disability and quality of life among PLHIV in Harare, Zimbabwe.
